# Accelerated peripheral nerve repair using surface-modified biomaterials for targeted capture of glial-derived growth factors post-neurorrhaphy

**DOI:** 10.1371/journal.pone.0319979

**Published:** 2025-04-11

**Authors:** Shiu-Jau Chen, Chih-Ming Lin, Chiung-Hui Liu, Yin-Hsiu Chen, Yu-Lin Hsieh, You-Cheng Lin, Yin-Hung Chu, Chung-Yao Ku, Wei-Li Liao, Wen-Chieh Liao

**Affiliations:** 1 Department of Medicine, MacKay Medical College, New Taipei, Taiwan; 2 Department of Neurosurgery, MacKay Memorial Hospital, Taipei, Taiwan; 3 Department of Neurology, Changhua Christian Hospital, Changhua, Taiwan; 4 Department of Post-Baccalaureate Medicine, College of Medicine, National Chung Hsing University, Taichung, Taiwan; 5 Doctoral Program in Tissue Engineering and Regenerative Medicine, College of Medicine, National Chung Hsing University, Taichung, Taiwan; 6 Department of Anatomy, Faculty of Medicine, Chung Shan Medical University, Taichung, Taiwan; 7 School of Post-Baccalaureate Medicine, College of Medicine, Kaohsiung Medical University, Kaohsiung, Taiwan; 8 Department of Anatomy, School of Medicine, College of Medicine, Kaohsiung Medical University, Kaohsiung, Taiwan; 9 Department of Medical Research, Kaohsiung Medical University Hospital, Kaohsiung, Taiwan; 10 Graduate Institute of Anatomy and Cell Biology, College of Medicine, National Taiwan University, Taipei, Taiwan; University of California Riverside, UNITED STATES OF AMERICA

## Abstract

Peripheral nerve injury (PNI) commonly leads to motor or sensory dysfunction, with nerve grafts being the standard treatment for neurorrhaphy. Despite advancements in biomaterials for nerve-tissue engineering, the rate of nerve regeneration remains slow. Therefore, this study aims to improve further the understanding of the impact of syndecan-3 (SDC3)-modified small intestine submucosa (SIS) on nerve reconstruction by employing two advanced approaches: cation recruitment and local growth factor delivery. Immunofluorescence staining confirmed the presence of SDC3 conjugated on the SIS. The enzyme-linked immunosorbent assay measured sustained glial cell line-derived neurotrophic factor (GDNF) levels in the SDC3-coated SIS. *In vitro* studies showed that SDC3-coated SIS retained GDNF in a dose-dependent manner, significantly enhancing Schwann cell proliferation compared with basal conditions. RSC96 cells proliferated most effectively on SDC3-coated SIS loaded with GDNF, and neuro-2A neurites were significantly longer on this material at 48 hours. One-month post-neurorrhaphy, morphological analysis revealed a 1.6 ±  0.02-fold increase in the number of β3-tubulin-positive axons in the GDNF+SDC3-coated SIS group compared to the SIS group. CMAP amplitude increases in the GDNF+SDC3-coated SIS group as more functioning motor axons are connected to the target muscle of ESN rats. This study provides valuable insights into the development of customized SDC3-coated SIS for promoting nerve tissue regeneration and accelerating rehabilitation.

## 1. Introduction

Choosing ECM-based biomaterials that mimic the neural environment is essential for achieving optimal biological performance. Small intestinal submucosa (SIS) exhibits rapid nutrient permeation, unique mechanical properties, resorbable capabilities, and low immunogenicity. Unlike synthetic scaffolds, SIS primarily consists of type I, III, IV, and VI collagen fibers, glycoproteins, glycosaminoglycans (GAGs), and proteoglycans (PGs), contributing to cell attachment and signaling [1,2]. Its heparan sulfate proteoglycan (HSPG) components contain high-affinity binding sites for various growth factors, enhancing cellular signaling in injured tissues.

SIS has been successfully applied in various clinical applications, including blood vessels, the peritoneum [3], the abdominal wall [4], the dura mater [5], the intervertebral disc [6] reconstruction, and nerve conduits [7]. It is eventually replaced by well-organized host tissues, ensuring seamless integration. However, the heterogeneous distribution of HSPG in SIS may vary depending on cell type and physiological state.

Focusing on SIS’s composition and clinical application, syndecan-3 (SDC3) emerges as a promising element. SDC3 is a transmembrane core protein that transports heparan sulfate (HS) and chondroitin sulfate (CS) chains [8]. Its anionic polysaccharides exhibit a strong affinity for positively charged growth factors, supporting axonal pathfinding and synaptic formation. Conjugating SDC3 to SIS and incorporating growth factors could enhance peripheral nerve regeneration (PNR). The electronegative components of SIS, particularly GAGs, attract positively charged growth factors such as basic fibroblast growth factor (bFGF), vascular endothelial growth factor (VEGF), and glial cell line-derived neurotrophic factor (GDNF) [9] via electrostatic interactions. GDNF, in particular, promotes neuronal survival, glial proliferation, and axonal integrity.

Finally, the negative charge of SDC3 in SIS makes it an excellent platform for growth factor retention and presentation, amplifying therapeutic potential for nerve regeneration. Our study evaluated SDC3-modified SIS combined with exogenous growth factors as a novel strategy to enhance PNR.

Using a rat model of end-to-side neurorrhaphy (ESN), we systematically investigated its effects on Schwann cell behavior, neurite outgrowth, and functional recovery. Results demonstrated that SDC3-modified SIS effectively modulates Schwann cell proliferation, supports neural cytoskeleton formation in regenerative tissues, and accelerates functional recovery.

These findings highlight the therapeutic potential of SDC3-modified SIS biomaterials as enhancers of neurotrophic factor delivery and nerve regeneration.

## 2. Materials and methods

### 2.1. Experimental animals, surgical procedures, and tissue preparation

Experimental animals: Young adult male Wistar rats weighing 200–300 g were purchased from the National Laboratory Animal Center, Taiwan. All experimental animals were housed under the same conditions with controlled temperature and humidity. The Chung Shan Medical University Laboratory Animal Center Authorities authorized the surgical procedure (IACUC Approval: 2594).

Use isoflurane anaesthesia (100–300 ml/min gas flow) with a vacuum device in the animal centre laboratory. Open the breathing regulator fully, place the animal inside, and wait until anaesthesia is achieved [10]. To reveal the left brachial plexus, a brief incision was performed along the left mid-clavicular line. The musculocutaneous nerve (McN) and the ulnar nerve (UN) were visible. To attach the cut end of McN with 10–0 nylon sutures under a surgical microscope, McN was transected at the pectoralis major muscle’s edge, and an epineurium window the size of McN was sliced open on the UN, being careful not to harm its contained axons. The wound will then be closed with 5–0 silk. Animals were allowed to recover for 30 days following surgery. Four groups of rats were created. A sham procedure involving only the brachial plexus was performed on Group I (n =  6). End-to-side neurorrhaphy was administered for 30 days to groups II (ES1M-SIS group, n =  6), groups III (ES1M-SDC3-coated SIS group), and groups IV (ES1M-GDNF+SDC3-coated SIS group). If animals show signs of suffering, Rimadyl MD (5-gram, tablets; bio-serve, Flemington, USA) will be provided for pain relief and nutrition. All experimental groups were sacrificed by transcardiac perfusion. For quantitative morphological analysis, half of the rats from all experimental groups were deeply anesthetized using Zoletil (40 mg/kg, intraperitoneally), ensuring a surgical plane of anesthesia. The animals were then subjected to transcardiac perfusion with 100 mL of Ringer’s solution to clear blood from the vessels. This was followed by perfusion fixation with 4% paraformaldehyde in 0.1 M phosphate buffer (PB, pH 7.4) for 45 minutes. To alleviate suffering, the animals were monitored during the procedure to confirm a lack of response to external stimuli, and humane endpoints were established and adhered to as necessary.

After perfusion, the repaired nerve was carefully dissected and immersed in the same fixative solution for another 2 hours to ensure adequate fixation. For immunoblotting analysis, the remaining half of the ESN animals were also deeply anesthetized with Zoletil (40 mg/kg, intraperitoneally) and perfused with Ringer’s solution. Subsequently, the musculocutaneous nerve was rapidly excised under a dissecting microscope to minimize ischemic damage. The dissected samples were immediately snap-frozen in liquid nitrogen and stored at –80 °C until further use.

### 2.2. Preparation of the decellularized sis membrane

**Fig 1:**
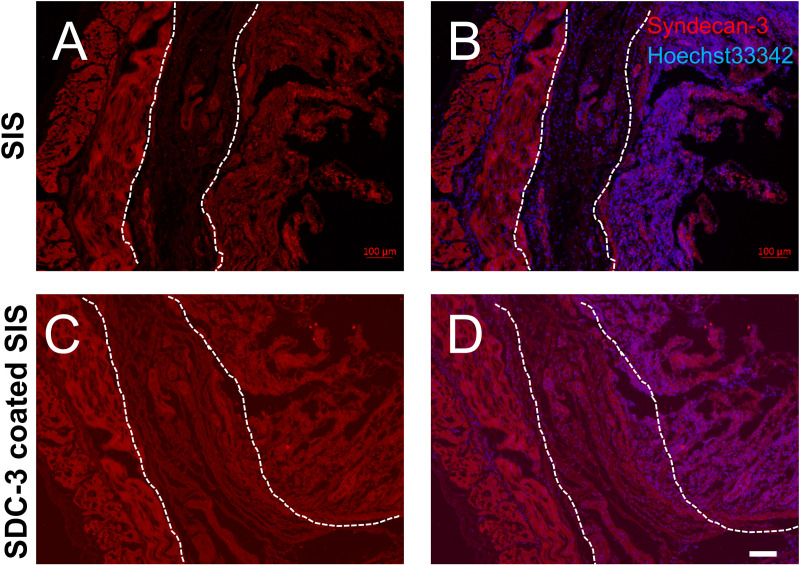
Syndecan 3 expression in the porcine small intestine. The microscopic appearance of SIS membrane. Histologic images showing the standard SIS (A and B) and the SDC3-coated SIS (C and D). The SIS was immunostained with anti-syndecan 3 antibody (red, cy3 fluorescent). Nuclei DNA was counterstained with hoechst33342 (blue; B and D). Note that standard SIS (A; dotted line area) and the SDC3-coated SIS (C; dotted line area), the submucosa layer of the small intestine, was coated with syndecan-3. Scale bars =  50 μm.

Landrace ×  Yorkshire ×  Duroc, or LYD, crossbred developing pigs, both sexes, 6 months of age, weighing an average of 130 ±  0.55 kg, were the source of the porcine jejunum section from Bioking Technology Co., Ltd. To obtain the target biomaterial, the submucosal layer, the mucosa layer was scraped off using a peeler. Following an alkaline and acid treatment, the pig small intestine submucosa (SIS) underwent a decellularization process. The materials were first mechanically cleaned and then incubated for 16 hours in a solution of 100 mM ethylenediaminetetraacetic acid (EDTA) (Sigma) in 10 mM sodium hydroxide (NaOH) with a pH of 11–12. Six hours were spent in the second incubation in 1 M hydrochloric acid (HCl) in 1 M sodium chloride (NaCl) at pH 0–1. After 16 hours of incubation in 1 M NaCl, any remaining compounds were removed with a 24-hour PBS wash. Every treatment was performed while being continuously shaken. The tissue was subsequently moved back to its initial orientation, and a cryostat microtome was used to gather 160 μm submucosa sections. Fig 1 displays the sis’s morphological image.

A freeze dryer (Model GLD-N137-K; ULVAC; Ningbo, China) was used to lyophilize the material for 24 hours at -80 °C. After being exposed to UV radiation for 30 minutes, the dry sis sheet was vacuum-sealed into hermetic packaging to sterilize it. The sis sheets were thawed as needed and kept in terminal storage at -80 °C. Then, using a rat model, we implanted the sis membrane as a neuroprotective membrane at the location of the nerve connection.

### 2.3. Surface modification of small intestine submucosa membrane

The cross-linking solution was prepared by dissolving N-(3-Dimethylaminopropyl)-N′-ethyl carbodiimide hydrochloride (EDC, Sigma) and N-hydroxysuccinimide (NHS, Sigma) in the presence of 2-(N-Morpholino) ethane sulfonic acid (MES) buffer to achieve final concentrations of 0.5 M each. The solution was mixed in a 1:1 ratio to form the activation medium. The hydrated SIS membranes were immersed in the crosslinking solution. Next, SDC3 (10 μg/mL, R&D Systems, Inc. Minneapolis, USA.) was added to the reaction mixture to enable conjugation with the SIS membrane [11]. The reaction was performed under gentle agitation for 2 hours at room temperature. After the crosslink reaction, the SIS membranes were washed extensively in phosphate-buffered saline (PBS) to remove unreacted EDC, NHS, and other residual components. A minimum of three washes, each lasting 5 minutes, were performed using fresh PBS. As needed, 2 µl recombinant GDNF protein (20 ng/µl; Pepro Tech, Inc. Rocky Hill, NJ, USA) was incorporated into the SDC3-coated SIS membrane through a 2-hour incubation. After being lyophilized and sterilized by 30 minutes of exposure to UV light, the resultant product was vacuum-sealed into hermetic packaging. As needed, the sis sheets were thawed from their terminal storage at –80°C. The SIS membrane was then implanted in a rat model as a neuroprotective membrane at the point of nerve connection.

### 2.4. Immunofluorescence stain

The collected tissue sections were immersed in a blocking solution containing 4% normal goat serum and 5% bovine serum albumin (Sigma-Aldrich, St. Louis, MO, USA) for 1 h to block non-specific binding. Following a phosphate-buffered saline (PBS) rinse, the sections were incubated for 18 hours at 4 °C in the blocking medium with anti-syndecan-3 (Santa Cruz Biotechnology, INC, mouse), anti-GDNF (Taiclone, Taipei, Taiwan, rabbit), and anti-β3-tubulin (Proteintech, rabbit) antibody. Cy3-conjugated anti-rabbit IgG (1:200, Jackson ImmunoResearch, West Grove, PA, USA) and Alexa Fluor anti-mouse IgG (1:200, Jackson ImmunoResearch, West Grove, PA, USA) antibodies were added to the sections and incubated for one hour after PBS washing. Lastly, Hoechst 33342 was used as a counterstain for the sections.

Following mounting, a confocal fluorescence microscope (SP8, Leica Microsystems, Wetzlar, Germany) was used to view the labeled tissues. A stacked sequence of scans of a SIS section (with a total thickness of 160-μm) were used to construct each confocal photomicrograph. Z-stack images were acquired from regenerated axons, using a 5-μm optical slice thickness with 10 z-sections recorded at 0.5-μm intervals.

### 2.5. ELISA protein quantification at each time point of a GDNF over a 7-day period

To verify the high expanses and instability of GDNF release from the biomaterials, the SIS or SDC3-coated SIS were soaked in GDNF solutions at different time points from day 1 to day 7. Then, the GDNF solution was collected and analyzed using enzyme-linked immunosorbent assays (BT Lab, Rat GDNF ELISA kit, Cat. No E0351Ra). The absorbance of the mixture will then be measured by ELISA reader at 562 nm. The optical density was determined by the ARS standard curve previously built (Myassay online software) [12].

### 2.6. Functional recovery tests: Compound muscle action potential recording

Compound muscular action potentials (CMAPs) of the regenerated nerves and target muscles were recorded using the Power Lab electromyogram (AD equipment, Sydney, Australia) to assess the functional state of a motor unit pool in ESN rats. To capture the data, a silver stimulating electrode was placed beneath the rejoined locations, while a silver recording electrode was inserted into the biceps brachii muscles. The recording and stimulating electrodes were placed one centimeter apart and were sutured with a 5–0 nylon thread. The animals’ tails were fastened to a ground wire. A 5 mA current was supplied along with a 0.2 ms square wave pulse that had a 0.2 Hz repetition frequency. The measured amplitudes were analyzed once the outcomes were digitally recorded.

### 2.7. Cell culture and treatments

Purchased from the Chinese Academy of Sciences’ Cell Bank, the rat Schwann cell line, RSC 96 cells, and neuro-2A cells were cultured in DMEM with 2 mm glutamine, 100 units/mL penicillin, 100 μg/mL streptomycin, and 5% fetal bovine serum (FBS) in 5% CO_2_ at 37°C for the *in vitro* cell biology study. The plating medium was supplemented with varying amounts of syndecan-3 (at concentrations of 1 and 10 μg/ml) and GDNF (at concentrations of 1 ng/ml) to examine the possible proliferative effects of these substances on Schwann cells. To examine whether the proliferative effects of heparan sulfate (HS) chains on syndecan-3 were mediated through of syndecan signaling, heparinase III treatment (R&D Systems, Minneapolis, USA) at the concentration of 0.5 mU/ml was added to confirm HS-GAG chain-induced GDNF mediated cell signaling pathway for 2 h at 37°C. After incubation, the cells were harvested and extracted for further analysis.

### 2.8. Cell counting kit-8 (CCK-8) assay

A colorimetric assay based on the conversion of tetrazolium salt (WST8) into an orange-colored formazan product was used to determine cell viability. In brief, RSC 96 cells were seeded into 96-well plates (10 µl/well) and cultured in 100 µl of culture media containing 5% FBS and various doses of syndecan-3. The extents of cell proliferation were measured after 24 and 48 hours of incubation using the Cell Counting Kit-8 (CCK-8; Sigma-Aldrich) assay for 4 hours at 37°C. To assess the number of live cells in each well, the absorbance at 450 nm was measured.

### 2.9. Western blotting

Lysates of neuro-2A cells in four groups was collected at day 3 post cell-seeding, being subjected to immunoblot analysis. The neuro-2A cells will first be dissolved with Kaplan buffer (50 mM Tris buffer, pH =  7.4, 150 mM NaCl, 10% glycerol, 1% NP40, and protease inhibitor cocktail). After centrifugation, the mixture containing desirable proteins will then be separated and electroblotted onto nitrocellulose membranes. Following blocking and antibody-probing process, proteins related to neurite outgrowth could be detected by ECL solutions and further analyzed by image J software.

### 2.10. Statistical analysis

Following the completion of the aforementioned investigations, one-way ANOVA and the Bonferroni post hoc test were used to analyze the data gathered from the immunofluorescence, immunoblotting, and spectrometry procedures in the sham-op and ESN rats. The Kolmogorov–Smirnov test must be used to verify the assumption of normalcy (P >  0.1). This supposition is tested by GraphPad Prism (PRISM, GraphPad Software, San Diego, CA, USA). P <  0.05 was regarded as a significant difference between the experimental group and the sham-operation group. The data were presented as mean ±  SD.

## 3. Results

### 3.1. Immobilization of HSPGs in the small intestine submucosa for biomedical applications

A standard approach for SDC3 immobilization to SIS was developed, employing an SDC3 solution) After SDC3 coating on SIS, immunofluorescence images show the standard SIS (Fig 1A and 1B) and the SDC3-coated SIS (Fig 1C and 1D). The SIS was immunostained with an anti-syndecan-3 antibody (red, Cy3 fluorescent). Nuclei DNA was counterstained with Hoechst 33342 (blue; Fig 1B and 1D). Note that standard SIS (Fig 1A; dotted line area) and the SDC3-coated SIS (Fig 1C; dotted line area), the submucosa layer of the small intestine, were coated with syndecan-3 successfully.

### 3.2. ELISA for released GDNF

**Fig 2:**
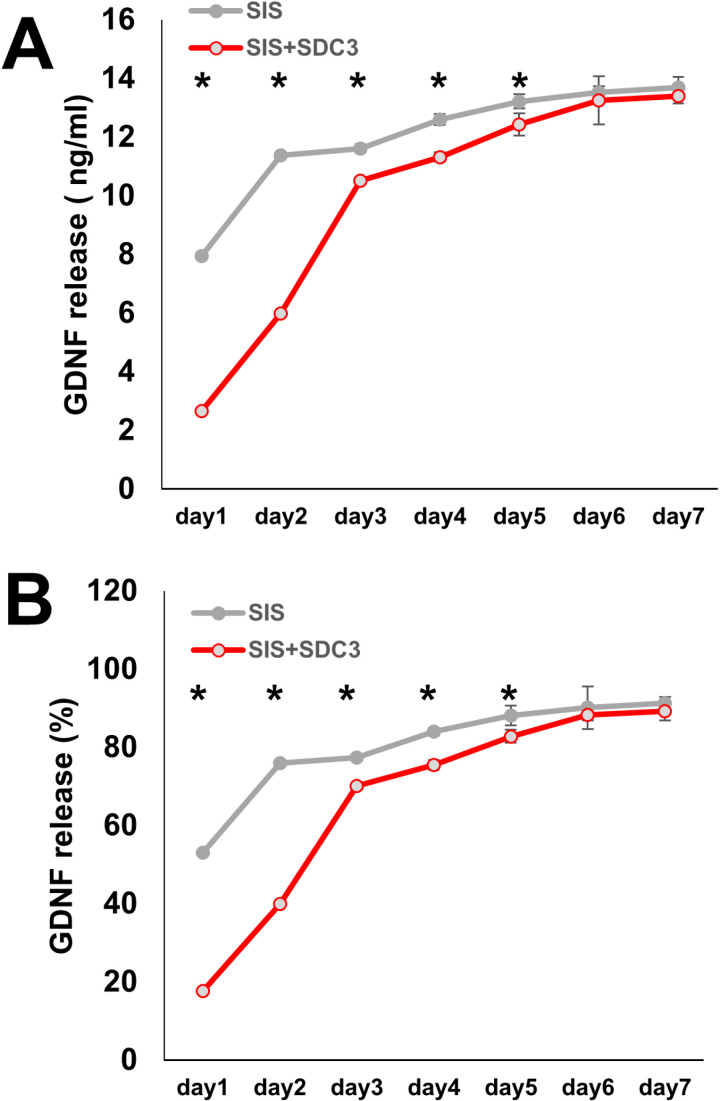
Release kinetics of GDNF from SIS with and without syndecan-3 (SDC3) added. (A) The cumulative release amount of GDNF from SIS and SDC3-SIS in a neutral condition (pH 7.4) at different time points from day 1-7. (B) *In vitro* release of GDNF from SIS and SDC3-SIS in a neutral condition (pH 7.4) for 7 days. Notes: SIS quantification of GDNF in SDC-3 SIS. Compared to SIS showed a relatively stable content of released GFs in DMEM, which then decreased greatly in the SIS after SDC-3 crosslinking. (*P <  0.05). Abbreviations: GDNF, glial cell-line derived neurotrophic factor; ELISA, enzyme-linked immunosorbent assay.

To evaluate the capacity of SIS-SDC-3 to stabilise the release of GDNF, we quantified the GDNF concentration at various time points from days 1–7. ELISA analysis of the SIS group indicated sustained GDNF release of 13.68 ±  0.05 ng/mL over the 7-day period, with an initial burst effect (11.38 ±  0.22 ng/mL) accounting for 76.97 ±  1.50% of the total release observed within the first two days (Fig 2A and 2B). In contrast, ELISA analysis of the SDC3-coated SIS group detected an initial GDNF release of only 5.98 ±  0.82 ng/mL, accounting for 43.81 ±  5.5% of the total release within the first two days (Fig 2A and 2B). These results suggest that the SDC3 coating modulates the release kinetics of GDNF, potentially influencing its bioavailability and therapeutic efficacy in tissue engineering applications.

### 3.3. HSPG-based SIS for growth factor delivery

**Fig 3:**
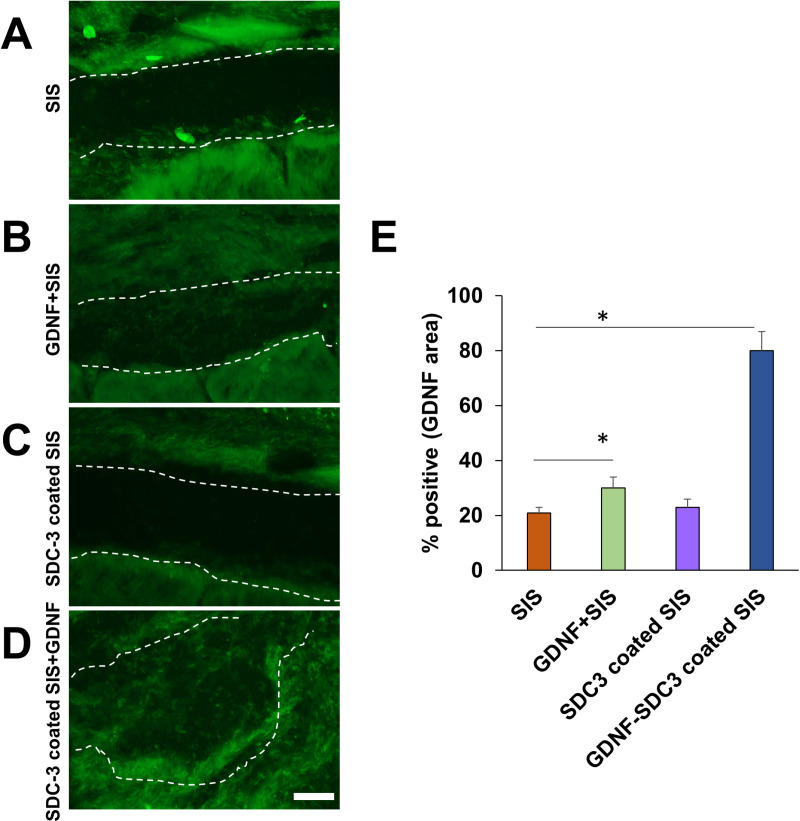
The assessment of GDNF retention within SIS. Immunofluorescence images showing GDNF in a control sis (A), GDNF-soaked sis membrane (B), SDC3-coated sis (C), and GDNF-SDC3-coated sis (D). The area bounded by the dot lines was the submucosa layer of the small intestine. The sis was immunostained with an anti-GDNF antibody (green). Scale bar =  200 μm. (E) The histogram showed the percentage of positive GDNF area. The percentage of GDNF area was higher in the GDNF-SDC3-coated sis when compared to that of the typical sis. Data are presented as mean ±  standard deviation from three independent experiments. * P <  0.05 compared to the control sis.

Immunofluorescence was used to determine the presence of GDNF. GDNF retention was examined by immunostaining for SIS with anti-GDNF antibodies after immobilising GDNF with SDC3 (Fig 3). Confocal micrographs revealed that GDNF expression was rarely observed in uncoated SIS membranes (Fig 3A). In contrast, GDNF was maintained at high levels throughout the SDC3-coated SIS (Fig 3D and 3E).

### 3.4. Analyzing the effect of HSPGs on SC proliferation

**Fig 4:**
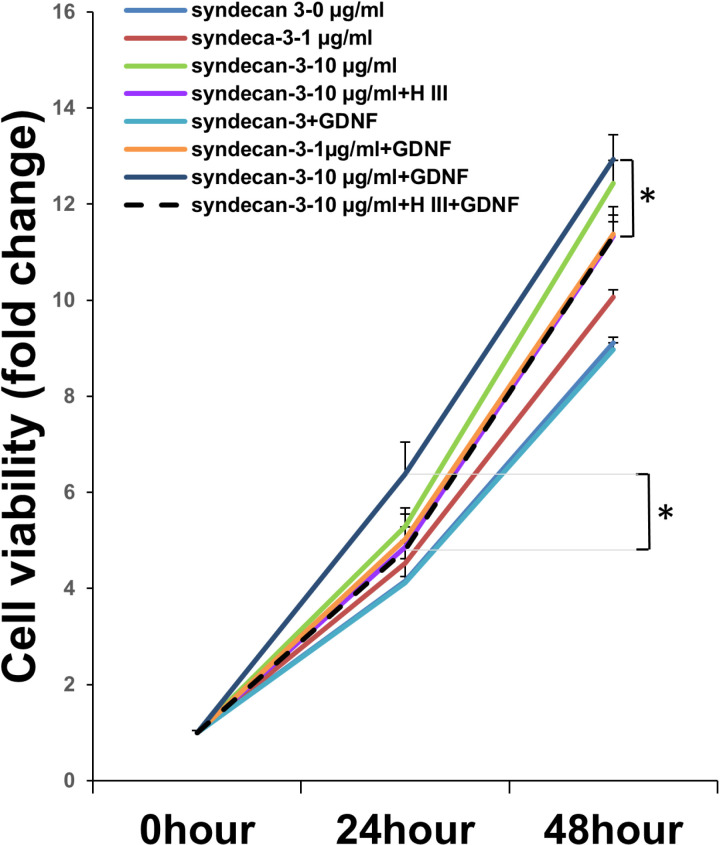
SDC3-coated small intestinal submucosa (SIS) scaffold enhances rat RSC96 Schwann cell (SCs) proliferation *in vitro.* Line charts showing the potential effects of SDC-3 coated SIS with GDNF on cell viability of RSC 96 cells. The cell viability was estimated by measuring the absorbance at 450 nm for the cell counting kit-8 (CCK-8) assay. Note that following 48 hrs. of RSC96 cell seeding on the membrane, the absorbance of CCK-8 was significantly increased with the maximal change observed in the concentration of 10.0 ug/ml of SDC-3 (dark blue line). Also note that in cell viability on SIS pretreated with heparinase III (HIII), the extent of cell viability was significantly suppressed, which suggested that the proliferative effects of GDNF were mediated by syndecan-3 in the GDNF signaling pathway. * P <  0.05 as compared to that of the group of 10 ug/ml of SDC-3 with GDNF 0.5 mU ml^* − *1^ heparinase III (black dot line).

The biological activity of the released GDNF was investigated by assessing its ability to stimulate the proliferation of RSC96 cells to confirm whether the growth factors released from SIS remained bioactive (Fig 4). RSC96 cells exhibited the lowest growth in basal medium without GDNF and with GDNF. Moreover, GDNF delivered from SDC3-coated SIS significantly improved RSC96 growth compared with GDNF addition in the basal medium and the basal medium alone at 48 h. Impressively, RSC96 cells exhibited poor proliferation in response to GDNF release from the SDC3-coated SIS at 48 h following heparinase III treatment. RSC96 exhibited the highest proliferation in an SDC3-coated (10 µg/ml) SIS with GDNF (1 µg/ml) at 48 h. These results indicate that the GDNF released from SDC3-coated SIS was superior to that released from the other treatments.

### 3.5. Analyzing the effect of HSPGs with growth factor on the neuro-2A cells differentiation

**Fig 5:**
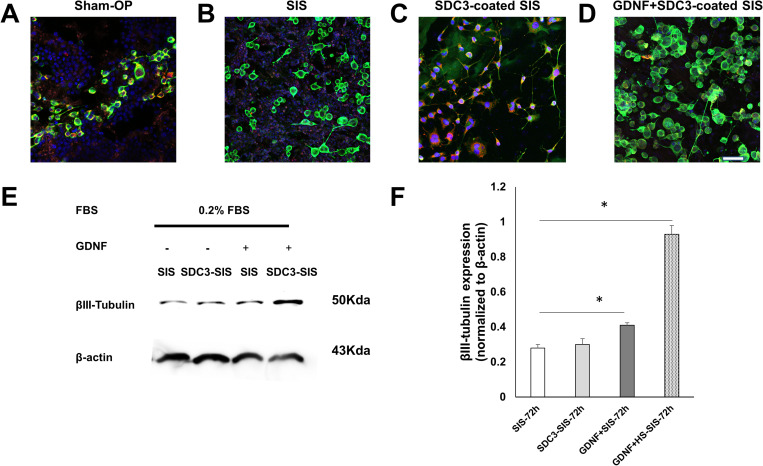
SDC3-coated small intestinal submucosa (SIS) scaffold enhances Neuro-2A cell differentiation *in vitro.* Immunofluorescent image showed neuro-2a cells were subjected to various culture conditions, namely SIS (A), SDC3 coated-SIS(B), GDNF+SIS (C), SDC3 bound GDNF-modified SIS (D) and then photographed with confocal microscopy at 20 × magnification. The SIS and neuro-2A cells were immunostained with anti-syndecan-3 (red-cy3fluorescence) and anti- ß3-tubulin antibodies (green-FITC fluorescence). The nuclei DNA was counterstained with hoechst33342 (blue). Please note that ß3-tubulin-positive neuro-2A cells (green) generated more neurites on SDC3-coated SIS (B) than that of neuro 2A cells on SIS membrane (A) at 48 hours. Scale bar =  50 μm. (E) shows individual levels of β3-tubulin presenting in neuro-2A cells 72 hours after seeding on SIS. Histogram (F) shows that β3-tubulin expression was higher in the GDNF+SIS-72h group than in the SIS-72h group. In the meantime, GDNF+SDC3-SIS-72h also showed higher β3-tubulin expression than that of the SIS-72h group. Data are presented as mean ±  standard deviation from three independent experiments. *P <  0.05 compared to the SIS-72h group.

The SIS with HSPG served as a scaffold for RSC96 cell proliferation. In this study, we investigated the effects of SDC3-coated SIS and GDNF on neuro-2A differentiation. Immunofluorescence staining showed the area of neurites of ß3-tubulin (+) cells in SDC3-coated SIS (10 µg/ml) SIS with GDNF (1 µg/ml) was significantly higher than in the SIS membrane at 48 h (Fig 5A and 5B). These results suggest that the SDC3-coated (10 µg/ml) SIS with GDNF (1 µg/ml) scaffold increases neuro-2A differentiation. We thus explored new strategies for surface HSPG modification of SIS membranes to enhance cellular signalling. Furthermore, the protein expression level of the β3-tubulin using immunoblotting (Fig 5E). Histogram quantified the protein levels of β3-tubulin in neuro-2A cells (Fig 5F). SIS with GDNF group caused a 1.4-fold increase in β3-tubulin expression compared to that of the SIS group. Due to the excellent affinity between syndecan-3 and GDNF, the best efficacy of HS-GAGs–GDNF interactions was performed in the SDC3-coated SIS with GDNF group. The SDC3-coated SIS groups had the highest β3-tubulin expression, increasing by 3.3-fold.

### 3.6. In vivo study

**Fig 6:**
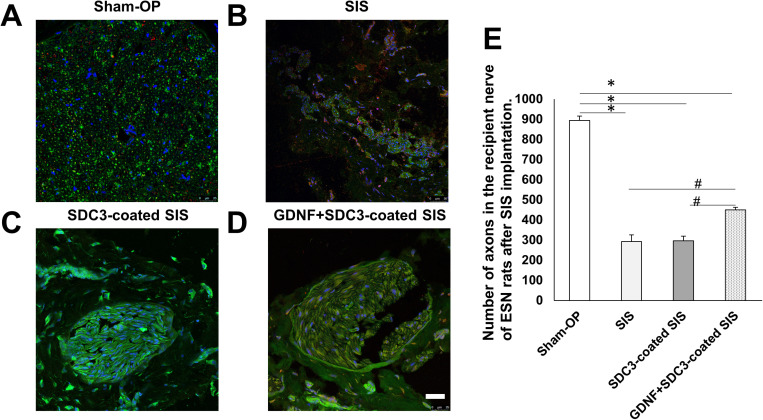
The effect of SIS and GDNF in combination on ESN rats. The confocal photomicrographs show the expression of β3-tubulin and F4/80 expression in the repaired nerve of the sham operative group (A), ES1M-rats with SIS (B), ES1M-rats with SDC3-coated SIS (C), and ES1M-rats with GDNF+SDC3-coated SIS (D). One month after the ESN, the recipient nerve tissue (the distal end of McN) was immunostained with anti-β3-tubulin (green) and anti-F4/80 (red). Following section labeling, Hoechst33342 (blue) was used as a counterstain for nuclei DNA. β3-tubulin-positive axons were observed in ES1M-SIS groups and SDC3-coated SIS group, while larger β3-tubulin-positive nerve fibers were arranged regularly, and fasciculus fibers in the ES1M-GDNF+SDC3-coated SIS group (D). Please note that F4/80-positive cells are found around the vessels. F4/80-positive cells were barely seen in epineurium 1 month after surgery. Scale bar =  50 μm. Histogram (E) shows an average number of sprouting axons in each group 1 months after SIS implantation. Data are present as mean ±  SD.

One month after surgery, regenerated axons were immunostained with anti-β3-tubulin to evaluate the morphological effects of GDNF combined with SDC3-coated SIS wrapped on the regenerating nerves of ESN rats (Fig 6). Immunofluorescence photomicrographs revealed scattered nerve fibre bundles in the SIS group (Fig 6B). However, in the SDC3-coated SIS group, most regenerating axons were rebuilt in large numbers (Fig 6C). Moreover, the GDNF+SDC3-coated SIS group exhibited thick axons arranged in a regular, high-density order (Fig 6D), demonstrating the most substantial regeneration among the four groups. F4/80 is a widely used marker expressed highly during active immune responses. Immunofluorescence micrographs demonstrated sporadic F4/80-positive cells (red) around blood vessels or interfascicular perineurium, indicating that SIS, SDC3-coated SIS, and GDNF+SDC3-coated SIS did not elicit severe inflammatory responses at the repair site. Our results showed that SDC3-coated SIS enhanced regeneration outcomes in ESN rats, such as increased fasciculus thickness and the number of nerve bundles. The combination of GDNF and SDC3-coated SIS demonstrated a synergistic effect, further improving axonal regeneration.

### 3.7. Functional recovery assessment via compound muscle action potential (CMAP) analysis

**Fig 7:**
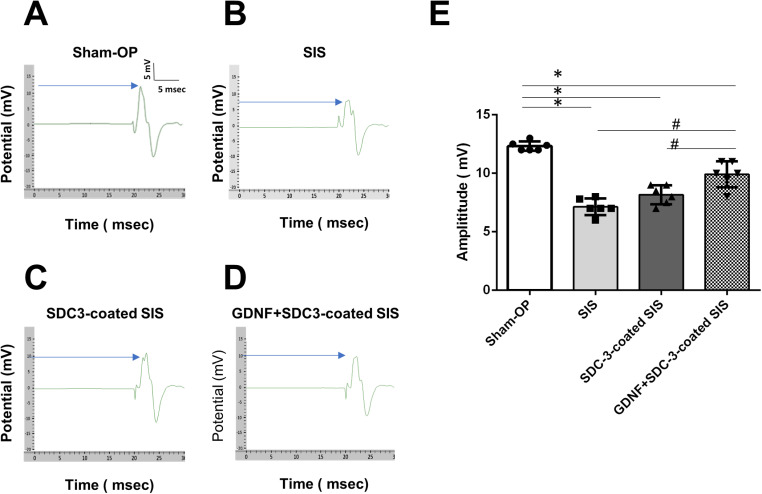
Compound muscle action potentials (CMAP) analyses for evaluating the functional recovery of ESN rats after SIS implantation. The recovery of CMAPs after ESN. The responses were recorded from the biceps brachii muscle upon activation of the nerve. The representative responses recorded the sham operative group (A), ES1M-rats with SIS (B), ES1M-rats with SDC3-coated SIS (C), and ES1M-rats with GDNF+SDC3-coated SIS (D) are illustrated. The arrow indicates the top of the amplitude. Stimuli at moderate (5 mA) strengths were applied to the nerve above the neurorrhaphy site. Bar-dot plots (E) show the averages of amplitude. *n* =  6 for each group. Values are the mean ±  standard deviation. *  *p* <  0.05 compared to that of the sham-operated value. ^#^ *p* <  0.05 compared to the corresponding ES1M rats after with GDNF+SDC3-coated SIS treatment.

We conducted CMAP analysis to validate the functional outcomes of the SDC3-coated SIS membrane on nerve regeneration in the ESN rat model. CMAP amplitude serves as a valuable indicator for monitoring and quantifying the progress of nerve regeneration (Fig 7).

The number of muscle fibres innervated by the regenerating nerves influences the amplitude parameters of the recorded CMAPs, providing an objective measure of experimental nerve recovery. CMAP responses were acquired by stimulating the repair site and recording the biceps brachii muscles.

Electrophysiological data revealed that all treatment groups exhibited lower CMAP amplitudes than the sham-op group, demonstrating an amplitude of 12.0 ±  0.03 mV. Notably, the GDNF+SDC3-coated SIS group demonstrated a significantly higher amplitude than the other treatment groups one month after ESN.

The SDC3-coated SIS group showed a larger amplitude of 10.0 ±  0.3 mV, followed by the SIS group with an amplitude of 7.0 ±  0.3 mV. Statistical analyses indicated significant differences between the GDNF+SDC3-coated SIS group and other treatment groups, suggesting superior functional recovery facilitated by the GDNF+SDC3-coated SIS membrane.

## 4. Discussion

It is challenging to develop effective nerve repair therapies for peripheral nerve injuries. Our findings revealed that SDC3 was the most significantly altered transmembrane HSPG in regenerated nerve tissue. At the same time, GDNF were reduced in the nerve tissue of ESN rats, with GDNF primarily produced by the reactive Schwann cell environment of the donor nerve [13].

SDC3 is known to be involved in peripheral nerve development, similar to regenerative nerve tissue [8]. However, when a robust growth factor is administered at the repair site, the tissue is likely unable to mount an effective regenerative response. We developed a therapy that could deliver missing GDNF to nerve tissues without requiring frequent injections to overcome this potential mechanism of growth factor loss.

Over the past few decades, innovations in tissue engineering techniques have emerged to mimic or protect the neural tissue microenvironment, thereby boosting nerve regeneration [14,15]. Controlling the release of growth factors (GFs) from biomaterials during real-time nerve regeneration is challenging. Previous studies on nerve cells have focused on the effects of growth factor delivery on neurite outgrowth because it is difficult to manipulate both physical characteristics and chemical linking [16]. This is the first study to report the management of GF release for nerve regeneration using an SDC3-modified SIS membrane, demonstrating the synergistic effects of GDNF and SDC3 in both *in vitro* and *in vivo* experiments.

GFs with positively charged amine groups bind strongly to electronegative elements in SIS, such as GAGs. It is essential to develop biomaterials with a higher electronegative charge to create an effective growth factor delivery scaffold. This helps mimic the natural interactions between GFs and ECM, leading to better control over how and where these factors act.

Our previous studies identified SDC3 in the ECM as a key regulator of the microenvironment at the nodes of Ranvier, particularly in attracting positively charged sodium ions [18]. Notably, SDC3 possesses five GAG attachment sites, exceeding those found in other syndecans [17,18]. By exploiting its electrostatic properties, SDC3 on the SIS membrane effectively gathers and stabilises essential molecules, such as GDNF, critical for the development and survival of spinal motor neurons [19].

Indeed, managing the sustained release and degradation rate of GDNF delivery carriers is challenging [20,21]. Many current techniques present obstacles to using low-affinity materials to deliver the GF payload. SIS provides an opportunity to increase the efficacy of pharmacological approaches in tissue engineering. In another strategy, some particular GFs are classified as heparin-binding, in which GFs connect with the endogenous ECM’s heparan sulfate proteoglycans with great affinity [22]. SDC3 coating and composite SIS exhibit a more robust association that keeps macromolecules such as GDNF in place.

SDC3 crosslinked with SIS forms a carrier with electrostatically retained GFs, serving as a nerve guidance conduit. The interplay between physical characteristics and chemical linkages enhances GDNF retention, enabling the SIS membrane to serve as an endogenous growth factor reservoir for on-demand release and support tissue healing [23,24].

In this study, SDC3-coated scaffolds for GDNF delivery influenced both neuronal and glial cell behaviours. SDC3-coated SIS induces neurite outgrowth in neuro-2A cells and RSC96 cell proliferation upon cell seeding. Through high-affinity binding of exogenous GDNF to the HS chains of syndecan-3 on the SIS surface, SDC3-modified SIS provides an optimal microenvironment that promotes Schwann cell proliferation and initiates neurite elongation. After peripheral nerve injury, Schwann cells exhibit an increased supply of GDNF, which promotes nerve regeneration [25–27]. Heparinase enzyme experiments demonstrated that heparan sulfate removal from SIS decreased the fold-change in the RSC96 cell proliferation assay, elucidating the important role of GDNF.

Results of our animal study with SIS implantation were consistent with the cell proliferation assay results, highlighting the optimal performance of the SDC3 (10 μg/mL) with GDNF and SDC3 (10 μg/mL) groups. SDC3-coated SIS with GDNF accelerated axon regeneration in ESN rats, providing critical evidence of the success of the nerve implant material. Furthermore, CMAP analyses corroborated the enhanced functional recovery observed in the GDNF+SDC3-coated SIS group compared with that in the SDC3-coated SIS group. These findings support the biochemical properties of syndecan-3, which can optimise the performance of SIS membranes in promoting nerve regeneration.

After three months of *in vivo* implantation, SIS naturally exhibited less than 10% degradation [28]. This aligns with our previous observation of minimal SIS membrane remnants at the surgical site after three months [7]. Utilising the timeline of SIS degradation, the effects of local growth factors can be eliminated when nerve regeneration is complete. Thus, using SIS as a nerve conduit can reduce the need for heparinase to degrade sulfated glycosaminoglycan chains from HSPG and avoid invasive injection.

## 5. Conclusion

Our findings demonstrated that SDC3-modified SIS enhanced the retention and bioavailability of GDNF through electrostatic interactions and cross-linking. The co-localisation of SDC3 and GDNF on the SIS resulted in a more pronounced neurogenic effect, as evidenced by robust axon extension. This approach holds significant potential for advancing therapeutic materials in peripheral nerve injuries and paves the way for further research in this promising field, inspiring new avenues for more effective treatments for patients with nerve injuries.

## Abbreviations

SDC3: Syndecan-3
ESN: End-to-side neurorrhaphy
GDNF: Glial cell-derived neurotrophic factor
HSPG: Heparan sulfate proteoglycan
GAGs: Glycosaminoglycans
ECM: Extracellular matrix.
